# Anchylosis of the Lower Jaw, Operation

**Published:** 1872-12

**Authors:** 


Anchylosis of the Lower Jaw; Recovery Through the Formation of a False Joint on Both Sides.—This case occurred in the same clinique as the preceding. The patient was twenty seven years old, and was affected with complete anchylosis of the jaw, the result of an inflammation following scarlet fever. The growth of both upper and lower jaw was appreciably retarded, in consequence of continued disuse Perfect relief was afforded by an operation, which consisted in removing a wedge-shaped portion of the lower jaw, the base of which pointed downwards. At the time of the report, four months after the operation, the motion of the jaw was completely restored. (Arch. f. Klin. Chirurgie, xiii. B. 3 H.)—*Boston Med. and Surg. Journal.*

